# Identification of cCMP binding and activated proteins

**DOI:** 10.1186/2050-6511-14-S1-P78

**Published:** 2013-08-29

**Authors:** Stefanie Wolfertstetter, Elisabeth Schinner, Franz Hofmann, Jens Schlossmann

**Affiliations:** 1 Department of Pharmacology and Toxicology, University of Regensburg, Germany; 2 FOR923, Institute for Pharmacology and Toxicology, TU, Munich, Germany

## Background

cAMP and cGMP are well established second messengers and essential for numerous of (patho)physiological processes. These purine cyclic nucleotides activate PKA and PKG, respectively. So far, there was no evidence of further cyclic nucleotides acting as second messengers. Meanwhile the existence of cCMP was described [1]. Formation of the cyclic pyrimidine nucleotide cyclic cytidine 3’,5’-monophosphate (cCMP) by cytidylyl cyclases is debated. cCMP induces relaxation of vascular smooth muscle via cGKI [2]. Furthermore, it was postulated that cCMP is relevant for cell growth [3] and blood cell function [4]. However, functions regulated by cCMP are mostly unknown.

## Results

Our aim is to identify cCMP-binding and -acitvated proteins and to elucidate whether cCMP plays a role as second messenger. We confirmed that cCMP activates the purified cyclic nucleotide-dependent protein kinases cAK and cGK. Then we investigated the effect of cCMP on purified cyclic nucleotide-dependent protein kinases and on intact tissues of wildtype (WT) and cGKI-knockout (KO) mice, namely jejunum, lung and brain. We identified various protein kinases as cCMP-binding proteins in tissue lysates. cCMP stimulated cyclic GMP-dependent protein kinases in WT tissue lysates, however there was no stimulation of phosphorylation in KO tissue lysates.

## Conclusion

These results indicate that cCMP could play a role in physiological processes in jejunum, lung and brain.
